# Macrolevel Analysis of Labour Productivity Losses Associated With Breast Cancer Among Women in 47 African Countries

**DOI:** 10.1155/ghe3/4330365

**Published:** 2025-07-15

**Authors:** Mustapha Immurana, Ibrahim Abdullahi, Kingsford Norshie, Elvis Reindolf Kale, Abdul-Aziz Iddrisu, Irene Honam Tsey, Evelyn Acquah, Maxwell Ayindenaba Dalaba

**Affiliations:** ^1^Institute of Health Research, University of Health and Allied Sciences, Ho, Volta Region, Ghana; ^2^Department of Economics, Memorial University of Newfoundland, St. John's, Newfoundland and Labrador, Canada; ^3^Emergency Unit, Ho Teaching Hospital, Ho, Volta Region, Ghana; ^4^Department of Banking Technology and Finance, Kumasi Technical University, Kumasi, Ashanti Region, Ghana

**Keywords:** Africa, breast cancer, LP

## Abstract

**Background:** Breast cancer remains one of the major diseases affecting women in the world. Relative to high-income settings, women in low-income settings such as Africa are less likely to be diagnosed with breast cancer and are more likely to die when they are affected by the disease. Apart from the negative health consequences of breast cancer, it could also reduce the labour productivity (LP) of the affected persons, at both the micro- and macrolevels. Nonetheless, empirical evidence on LP effects of breast cancer are scant and mostly focused on the microlevel and, hence, do not provide broader insights into the productivity losses associated with the disease. This study, to the best of our knowledge, therefore, provides the first cross-country macrolevel empirical evidence of the effect of breast cancer (among women) on LP in Africa.

**Methods:** The study uses data on 47 African countries spanning the period 1992–2021. Disability-Adjusted Life Years (DALYs) associated with breast cancer in women is used as the baseline measure of breast cancer, while Years Lived with Disability (YLDs) and deaths associated with the disease in women are used as robustness measures. The system Generalised Method of Moments (GMM) regression is used as the main estimation technique, while two other estimators are used for robustness purposes.

**Results:** Our analysis reveals a negative statistically significant association between breast cancer DALYs and LP. Specifically, we find a percentage increase in breast cancer DALYs to be associated with a 0.27% and 0.87% fall in LP in the short- and long-run periods, respectively, at the 1% level of significance. The findings are robust using the other measures of breast cancer and different estimation techniques.

**Conclusion:** There is a need to enhance measures towards breast cancer prevention and control in Africa such as timely diagnosis, all-inclusive management of breast cancer, health promotion geared towards early detection and the creation of dependable referral systems to significantly reduce its associated LP losses.

## 1. Introduction

Breast cancer occurs when anomalous breast cells grow uncontrollably, forming tumours. If left untreated, these tumours can metastasise to other parts of the body which can be fatal. Nearly 99% of breast cancers occur among women [1]. For instance at the global level, in 2022, 2.3 million women were diagnosed with breast cancer, and 670 thousand deaths were recorded [1].

There are inequities in the burden of breast cancer among women. Hence, relative to developed countries, women in less developed countries are less likely to be diagnosed with breast cancer but are more likely to die if they get the disease [1]. Thus, low- and middle-income regions such as Africa bear a substantial burden of breast cancer. In sub-Saharan Africa for instance, in 2021, 731,970 females were diagnosed with breast cancer of which 60,133 died [2]. Moreover, among women 20 years and above, the breast cancer Disability-Adjusted Life Years (DALYs: sum of Years of Life Lost (YLLs) as a result of breast cancer premature mortality and years of healthy life lost as a result of breast cancer disability (i.e. Years Lived with Disability (YLDs) [3]) were nearly 2.057 million years [4].

Since health is a major constituent of human capital [5], morbidity and mortality caused by breast cancer can reduce labour productivity (LP) due to presenteeism and absenteeism. This issue is important for Africa, where a significant number of the population are females [6]. Thus, to advocate for increased resources towards fighting breast cancer on the African continent, it is important to provide not only cross-country evidence of the clinical burden of the disease but also its economic burden as well.

While some studies have examined the productivity losses associated with breast cancer [7–23], to the best of our knowledge, among these studies, only the study by Mbonigaba and Akinola [23] was devoted to an African country (i.e. Nigeria). Moreover, majority of these studies were focused on one country, while some of them (including the one in Nigeria) [23] were devoted to household or individual LP effects and not country-wide or continental (macro) level effects. These gaps tend to overshadow the wider LP losses associated with breast cancer as well as limiting the generalisations of the findings, especially on the African continent.

To this end, this study fills the gaps in the literature by examining the macrolevel effect of breast cancer among women on LP in several African countries. Doing so provides more broader insights on the LP losses associated with breast cancer among women in Africa, which are expected to increase investments and other efforts geared towards reducing the burden of the disease on the continent.

### 1.1. Brief Conceptual and Theoretical Framework of the Breast Cancer and LP Nexus

The human capital model of demand for health postulates health as a major component of human capital [5], which is a major driver of economic growth per the augmented Solow growth model [24]. The implication is that human capital is a significant determinant of LP. Hence, when people are sick, they become both physically and mentally weak, which negatively affect their productivity and vice versa [25].

Breast cancer can therefore reduce or curtail the participation of affected people in economic activity as a result of disabilities, morbidity and mortality. Moreover, family members or caregivers of breast cancer patients may have to abandon their work to take care of their sick relatives or friends. These will therefore culminate into a fall in LP at both the micro- and macrolevels.

**Figure 1:**
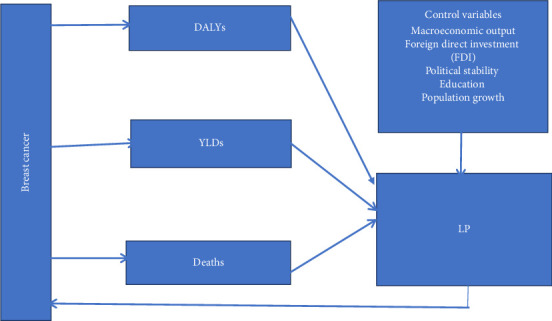
Conceptual framework of the relationship between breast cancer and LP. *Source:* Authors.

On the other hand, highly productive labours are more likely to earn higher, which could increase their ability to afford health care (both curative and preventative), hence decreasing their likelihood of contracting breast cancer. The conceptual link between breast cancer and LP can be found in Figure 1.

Aside from breast cancer, factors such as foreign direct investment (FDI), education, population growth, macroeconomic output and political environment can also affect the level of LP in a country. For instance, foreign firms could have different features relative to local firms; hence, FDI could affect LP [26]. In addition, since education and population growth are also forms of human capital [5, 24], they could affect the level of LP. Details of the expected signs of these variables can be found in the methods section.

## 2. Methods

### 2.1. Study Design, Data and Variables

**Figure 2:**
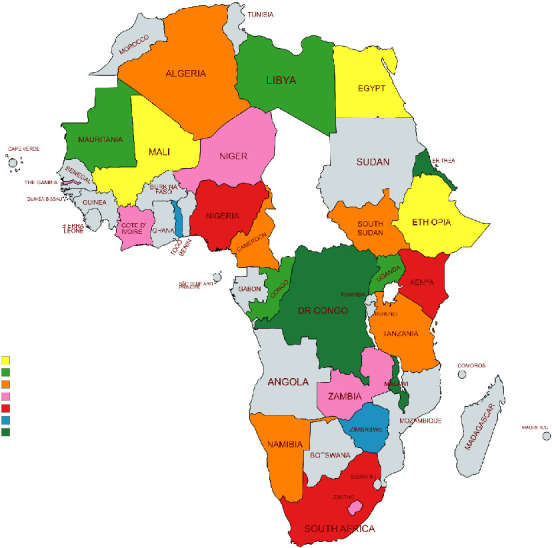
Map of selected countries. *Source:* The map is created by authors using mapchart.net [30].

A panel design is employed for this study because of the number of countries and the study period. Specifically, the study involves 47 countries in Africa (see Figure 2) with data over the period 1992–2021. The number of countries and the study period are dictated by the availability of data from all sources of data mentioned below. The dependent variable is LP, and the independent variable is the burden of breast cancer among women aged 20+ (BR). We use three indicators to measure the burden of breast cancer: in the baseline, we use DALYs associated with breast cancer while for robustness checks, we use YLDs and the number of deaths (Deaths) due to breast cancer as additional indicators. In particular, we employ DALYs because they are used to assess the overall burden of a disease since they combine both YLLs and YLDs. Thus, by employing DALYs, the burden of diseases with low disability but could cause premature mortality can be compared with the burden of diseases associated with disability but very little or no deaths [3]. The control variables used are macroeconomic output, FDI, political stability, education and population growth. Data on LP are obtained from the International Labour Organisation (ILO) [27]; data on the burden of breast cancer indicators are sourced from the Global Burden of Diseases Study (GBD) database [28]; and data on all the remaining variables are obtained from the World Development Indicators (WDI) of the World Bank [29]. Gaps in the data are filled using linear interpolation.

LP is measured by output (real or constant gross domestic product (GDP) in 2015 United States Dollars (USD)) per worker. DALYs and YLDs are measured in years, and the deaths are measured in numbers. Macroeconomic output is measured by constant GDP in 2015 USD, FDI is measured as net inflows in terms of balance of payments (in current USD), and political stability is measured by the perceptions on political stability as well as the absence of terrorism or violence ranked from 0 to 100. Education is measured as gross primary school enrolment, while population growth is measured by the annual growth rate in the number of people residing in a country. Both education and population growth are measured in percentages. All these definitions/measurements are from the aforementioned sources of data.

Given that illness will negatively affect the involvement of people (human capital) in production [25], we expect all the measures of breast cancer to have negative effects on LP. Since higher macroeconomic output is likely to be associated with higher competition among firms and infrastructural advancement, we expect it to have a positive effect on LP. Similarly, an enhancement in net FDI inflows is likely to be associated with technological advancement and transfer of skills [31]. We therefore expect FDI to have a positive effect on LP. A rise in political stability will provide safe environments for people to work, hence, increasing their productivity. Given that education enhances the efficiency of human capital [5], we expect a positive association between education and LP. Last, but not the least, higher population will increase the availability of labour; hence, we expect a positive association between population growth and LP.

### 2.2. Model and Estimation Techniques

To examine the effect of breast cancer among women on LP, we specify equation (1) below:

(1) $$\begin{array}{c} \text{LP}=f\left(\text{BR},X\right), \end{array}$$

where *X* is a vector of the control variables, and the rest of the notations are as already defined.

To capture the persistence of LP over time, we introduce the first lag of LP (LP_it−1_) as an independent variable (dynamic term) and respecify Equation (1) in a more estimable form as follows:

(2) $$\begin{array}{c} {\text{LP}}_{\text{it}}=\beta +{Ϫ\text{LP}}_{\text{it}-1}+{\delta \text{BR}}_{\text{it}}+ΩX_{\text{it}}+\varpi_{t}+\varepsilon_{\text{it}}, \end{array}$$

where *t* and *i* represent time (year) and countries, respectively. The intercept, time fixed effects (FE) and the disturbance or error term are, respectively, represented by β, *ϖ* and ε. Meanwhile, Ϫ,  δ and Ω are the coefficients of their associated variables.

Given the nature of Equation (2), there are two main estimation challenges that should be handled. First, the dynamic term could correlate with the error term leading to endogeneity [32]. Second, LP is likely to affect some of the right-hand-side variables leading to endogeneity. For instance, highly productive workers are more likely to earn higher, making them more capable of affording preventive care to avoid being sick, hence reducing their likelihood of contracting breast cancer.

Given the above endogeneity concerns, this study employs the dynamic panel system Generalised Method of Moments (GMM) regression as the main estimation technique because of its ability to deal with endogeneity. The system GMM addresses endogeneity using internal instruments, as well as level and first difference equations. However, for the system GMM estimates to be acceptable, there should be no second-order serial correlation and overidentification, and the number of instruments should be less than the number of cross sections (i.e. countries) to avoid the proliferation of instruments. Therefore, the Arellano–Bond (AB) and Hansen (H) tests are used to confirm the absence of second-order serial correlation and overidentification, respectively, when their respective *p*-values are insignificant [33–35].

In running the system GMM, we take 5-year average of the data to make them more suitable for the system GMM estimation and to smoothen or curtail the aberrations associated with recurrent data [33, 36]. Also, apart from FDI, political stability and population growth that are not log-transformed because they have either zero or negative values, all the remaining variables are subjected to log transformation. Such log transformation facilitates a reduction in the variations in how variables are measured as well as enabling the interpretation of coefficients as elasticities [37, 38]. Since Equation (1) is a short-run model, indicating the immediate effects of the independent and control variables on LP [39], we use the technique of Papke and Wooldridge [40] to derive long-run estimates (i.e. cumulative effects of the independent and control variables on LP [39]).

### 2.3. Further Robustness Checks

To further check the robustness of our estimates, especially the effect of breast cancer on LP, we use the nonaveraged data to run random effects (RE) and FE regressions in nondynamic models.

## 3. Results

In this section, we present summary statistics of the study variables and the system GMM regression estimates of the effect of breast cancer on LP in the sampled countries. We also present results of robustness checks using RE and FE regressions.

### 3.1. Summary Statistics of Variables

From Table 1, on average, LP; thus, GDP per worker is $6690.72. The average DALYs and YLDs associated with breast cancer are 29,486.18 years and 930.78 years, respectively, and the average number of associated breast cancer deaths is 856.43 people.

The average macroeconomic output over the period is $37.63 billion. In addition, the average net FDI inflows in terms of balance of payments, political stability rank, educational enrolment and population growth rate are −618.6 million, 33.99, 96.12% and 2.33%, respectively (Table 1).

### 3.2. System GMM Regression Estimates

In this subsection, we present the system GMM regression estimates of the effect of breast cancer on LP losses. All our estimates are free from overidentification, second-order serial correlation and the proliferation of instruments. Moreover, the F-stat. *p*-values indicate that our estimates have good fit (Table 2).

In the short-run, we find the past year's value (first lag) of LP to have positive statistically significant effects on the current level of LP at 1% level of significance in both the baseline (Model 1) and robustness models (Models 2 & 3) (Table 2).

Regarding the burden of breast cancer in women, we find the baseline measure (DALYs) to have a negative statistically significant effect on LP at 1% level of significance. Specifically, a percentage increase in breast cancer DALYs among women is found to decrease LP by 0.27%. Using the robustness measures of breast cancer (i.e. YLDs and Deaths), the results are not qualitatively different. Specifically, a percentage increase in breast cancer YLDs and Deaths among women is found to decrease LP by 0.18% and 0.25%, respectively, at 1% level of significance (Table 2).

Focusing on the control variables, in the baseline, we find a percentage increase in macroeconomic output to be associated with a 0.34% increase in LP at 1% level of significance (Model 1). Similarly, in the robustness estimates, a 1% increase in macroeconomic output is found to increase LP by 0.25% and 0.32% in Model 2 and Model 3, respectively, at 1% level of significance. For FDI, we find it to have a negative significant (at either 1% or 5% or 10% level) association with LP in all the models. Political stability is also found to have a positive significant association with LP (coefficient: 0.004, *p* < 0.01 (Model 1); coefficient: 0.005, *p* < 0.01 (Model 2); coefficient: 0.004, *p* < 0.01 (Model 3)). Similarly, a percentage increase in school enrolment (Education) is found to increase LP by 0.22% (Model 1), 0.14% (Model 2) and 0.24% (Model 3) at 1%, 10% and 1% level of significance, respectively, while the effect of population growth is found to be negative and significant (coefficient: −0.05, *p* < 0.05 (Model 1); coefficient: −0.05, *p* < 0.1 (Model 3)) (Table 2).

In the long-run, the effect of breast cancer in women on LP is negative and significant, which is like the short-run results. Thus, in the baseline, a 1% increase in breast cancer DALYs in women is associated with a fall in LP by 0.87% at 1% level of significance. Employing the robustness measures of breast cancer in women, we find that 0.85% and 0.84% decrease in LP are associated with a percentage increase in breast cancer YLDs and Deaths among women, respectively, at 1% level of significance (Table 3).

For the control variables, in the long-run, their signs and significance are like that of the short-run estimates (Table 3).

### 3.3. RE and FE Regression Estimates (Additional Robustness Checks)

Using the RE and FE regressions to check the robustness of our system GMM estimates, especially regarding the burden of breast cancer in women, we find the effects of DALYs, YLDs and Deaths on LP to be similar to the system GMM estimates (see Appendix Tables A1 and A2). Thus, RE and FE estimates confirm the robustness of the system GMM estimates of the negative significant effect of breast cancer in women on LP.

## 4. Discussion

This study, to the best of our knowledge, provides the first macrolevel empirical evidence of the effect of breast cancer (among women) on LP across African countries, which is important for unearthing the broader LP losses associated with the disease. Our baseline findings show that a percentage increase in breast cancer DALYs is associated with a 0.27% and 0.87% fall in LP in the short- and long-run periods, respectively. The findings are robust using different measures of breast cancer and estimation techniques. Using the average LP (output per worker) of $6690.715 ($6690.72) for our sampled countries (Table 1), the implication is that, when breast cancer DALYs increases by 1%, it decreases output per worker by $18.06 and $58.21 in the short- and long-run periods, respectively. These figures would translate into huge losses bearing in mind the number of women that are directly affected by breast cancer on the African continent, who may be less effective at work or unable to work again as well as those who may have to abandon their jobs to provide care or support to those suffering from the disease. For instance, in Germany, the United Kingdom (UK) and United States (US), 12% and 13% of caregivers of breast cancer patients were found to have reduced working hours and stopped working, respectively, after they started providing care [41]. Moreover, in low- and middle-income countries, caregivers of breast cancer patients have been found to encounter psychosocial challenges [42] which could negatively affect their productivity.

Our findings on the productivity losses associated with breast cancer are in tandem with those of Pycroft and Vasilev [18] who found that in the short-run, a LP (per worker) loss of -$1711 was associated with a 10% increase in the lagged rates of cancer in the UK. Similarly, Yin et al. [8] found $24,166 and $30,666 to be the annual value of missing work due to nonmetastatic and metastatic breast cancer, respectively, among women aged 18–64 years in the US. In another study in the US, the yearly average productivity loss associated with breast cancer was estimated to be $120,404 [10]. In Spain, it was found that premature breast cancer deaths among women was associated with 10.45% of total female LP losses from 2005 to 2014 [14]. Further, in Poland, breast cancer productivity losses were found to be €583.7 million and €699.7 million in 2010 and 2014, respectively, accounting for about 0.162%–0.171% of GDP over the period [16]. Additionally, in a cross-sectional study of women diagnosed with breast cancer in South-West Nigeria, it was found that an increase in the number of breast cancer cases reduced productivity by 19.5% [23]. Notwithstanding, the differences in our estimates of LP losses relative to these past studies could be attributed to the differences in the study design, countries used as well as the measurement of variables. For instance, none of these studies were devoted to several African countriesas done by our study. Moreover, in low-income settings such as Africa, due to less robust health infrastructure and systems, there is the likelihood of challenges with managing breast cancer cases effectively, which could lead to higher burden estimates, hence productivity losses.

Despite the significant LP losses associated with breast cancer in Africa, there appears to be low awareness, infrequent screening or examination, missing and late detection of cases and less equitable access to health care for those affected by the disease. It is therefore not surprising that, relative to high-income settings, women in low-income settings such as Africa are less likely to be diagnosed with breast cancer but are more likely to die upon diagnosis [1].

To reduce the burden of breast cancer towards achieving the World Health Organisation (WHO) Global Breast Cancer Initiative target of decreasing breast cancer associated deaths in the world by 2.5% annually, consequently preventing 2.5 million breast cancer mortalities in the world between 2020 and 2040 [1], there is the need to intensify efforts towards fighting breast cancer both in the short- and long-term [1]. Specifically, in the short-term, less costly initiatives such as timely diagnosis, all-inclusive management of breast cancer and health promotion geared towards early detection can be embarked upon.In the long-term, relatively high-cost interventions such as creation of dependable referral systems from facilities providing primary care, to facilities at the secondary level, and finally to devoted cancer centres, should be prioritised [1]. These can be achieved by increasing investment from both private and public sectors towards establishing mobile clinics with the capacity to offer breast cancer services that are equitably distributed, in the short-term, while ensuring the training of more health professionals specialised in breast cancer care, building of more health facilities as well as implementing health financing schemes that will greatly reduce out-of-pocket costs associated with breast cancer care in the long-term. Given resource constraints albeit several challenges including health, governments should prioritise the integrated management of noncommunicable diseases (NCDs) by implementing the WHO package of essential NCDs interventions which identifies approaches for managing a number of diseases including cancer, diabetes, cardiovascular diseases and chronic respiratory diseases. Doing so would ensure the efficient use of limited resources in fighting NCDs such as breast cancer [43].

As regards the control variables, the finding on the negative significant association between FDI and LP is surprising because FDI is expected to be associated with the transfer of technology and skills [31] that will boost LP. The finding on FDI conflicts with some past studies [26, 44, 45]. Nonetheless, FDI could be associated with acquisitions and mergers that may put pressure on workers regarding the fear of losing their jobs [46], as well as actual job displacement (especially through crowding out domestic investment [47]) which could negatively affect their productivity. For macroeconomic output, its positive association with LP is not surprising because rising macroeconomic output would be associated with higher competition among firms and the acquisition of equipment as well as other facilities which would boost LP. Moreover, higher macroeconomic output implies output per worker is high. The finding on the effect of macroeconomic output is similar to those of Moyo et al. [48] who found macroeconomic output (GDP growth) to have a positive effect on total LP in the Eastern Cape Province of South Africa. A similar outcome has been found among a sample of Organisation for Economic Co-operation and Development (OECD) countries [49].

When the environments in countries are politically stable, it ensures continued production by firms as well as the active participation of labour force in production without any distraction. It is therefore not surprising that we find a positive association between political stability and LP. Also, since education is expected to be associated with higher skills or efficiency [5], it is not surprising that we find a positive association between educational enrolment and LP. This finding concurs with Pycroft and Vasilev [18] who found a rise in education expenditure per worker to have a positive significant effect on LP in the UK as well as Emako et al. [45] who found education (human capital) to have a positive effect on LP among a sample of developing countries.

Our findings on the negative association between population growth and LP is surprising because higher population is expected to increase the labour force, hence LP. However, the negative association could be that, when there is population growth but skill mismatch (overskilled and underskilled), it will be detrimental to LP. For instance, among a sample of African countries, it has been found that 17.5%, 28.9%, 8.3% and 56.9% of employed youth were overskilled, underskilled, overeducated and undereducated, respectively. In addition, overskilled and overeducated youth were found to be less satisfied with their jobs as well as had a higher likelihood of on-job search [50], which could reduce their productivity.

## 5. Limitations

Notwithstanding our attempt to provide the first macrolevel empirical evidence of the effect of breast cancer (among women) on LP across African countries, this study is not without limitations. First, due to data paucity, we do not use all the countries on the African continent which could limit the extension of findings to be representative of Africa, although we believe that using 47 out of 54 countries is representative enough. Second, the source of data for the breast cancer indicators normally has paucity of primary data for low-income settings such as Africa. Although this gap is addressed during modelling, having more and befitting primary data would greatly help in improving the estimates [51]. Third, although breast cancer could affect men, this study only focuses on women because they comprise majority of the cases. Fourth, while our study is purely quantitative, adding a qualitative component could have provided further insights. Fifth, our study does not also look at how factors such as access to medical care and health expenditure could mediate the relationship between breast cancer and LP. Future studies may therefore focus on addressing some of these limitations if possible. In particular, future studies may consider cross-country qualitative studies to explore the insights of breast cancer patients and their employers and caregivers regarding the nexus between the disease and LP in Africa. Such studies may also look at the mediating roles of medical care and health expenditure in the breast cancer LP nexus by using quantitative data across African countries.

## 6. Conclusion

Breast cancer affects a significant number of women on the African continent. Apart from the health consequences, the disease could reduce the LP of the affected persons and their caregivers at both the micro- and macrolevels. However, past studies have mostly been single country in nature focused on the microlevel, with no cross-country macrolevel analysis devoted to the African continent. Doing a macrolevel analysis across several countries is important for unearthing the broader LP losses associated with the disease as well as increasing attention on the need to enhance efforts towards tackling it. This study, to the best of our knowledge, therefore, provides the foremost empirical macrolevel evidence of the effect of breast cancer (among women) on LP across Africa using 47 countries. Our findings reveal a negative significant association between breast cancer DALYs and LP in both the short- and long-run periods, and these findings remain robust using different indicators of breast cancer and estimation techniques. There is therefore the need to enhance frequent examination or screening, as well as universal access to less costly breast cancer health care to prevent and reduce the burden of the disease.

## Figures and Tables

**Table 1:** Descriptive statistics of variables.

Variable	Obs	Mean	Std. dev.	Min	Max
LP	282	6690.715	8221.759	479.93	58,025.09
DALYs	282	29,486.18	50,898.803	126.266	472,378.56
YLDs	282	930.778	1700.094	3.442	13,249.916
Deaths	282	856.428	1521.345	4.036	14,120.77
Macroeconomic output	276	3.763e + 10	7.528e + 10	1.253e + 08	5.031e + 11
FDI	258	−6.186e + 08	1.343e + 09	−8.755e + 09	4.443e + 09
Political stability	279	33.985	22.498	0	86.844
Education	271	96.12	23.318	26.687	155.12
Population growth	282	2.333	0.904	−2.171	4.87

*Note:* Averaged data are used; variables are not log-transformed.

**Table 2:** Short-run two-step system GMM estimates of the effect of breast cancer on LP.

	Model 1	Model 2	Model 3
L. Labour productivity	0.6883^∗∗∗^	0.7860^∗∗∗^	0.6995^∗∗∗^
	(0.0480)	(0.0266)	(0.0487)

DALYs	−0.2723^∗∗∗^		
	(0.0590)		

Macroeconomic output	0.3408^∗∗∗^	0.2504^∗∗∗^	0.3225^∗∗∗^
	(0.0649)	(0.0358)	(0.0638)

FDI	−0.0000^∗^	−0.0000^∗∗∗^	−0.0000^∗∗^
	(0.0000)	(0.0000)	(0.0000)

Political stability	0.0037^∗∗∗^	0.0048^∗∗∗^	0.0041^∗∗∗^
	(0.0007)	(0.0008)	(0.0008)

Education	0.2236^∗∗∗^	0.1352^∗^	0.2427^∗∗∗^
	(0.0714)	(0.0722)	(0.0750)

Population growth	−0.0482^∗∗^	0.0001	−0.0452^∗^
	(0.0233)	(0.0174)	(0.0253)

YLDs		−0.1829^∗∗∗^	
		(0.0284)	

Deaths			−0.2523^∗∗∗^
			(0.0563)

Constant	−3.7458^∗∗∗^	−3.6977^∗∗∗^	−4.6034^∗∗∗^
	(0.5970)	(0.6233)	(0.7922)

Observations	214	214	214
No. of countries	47	47	47
No. of instruments	36	36	36
AB	−0.3073	−0.3699	−0.2951
AB *p* value	0.7586	0.7115	0.7679
H	25.7690	27.6688	25.3617
H *p* value	0.3650	0.2743	0.3863
F-stat.	204,417.2013	233,286.2761	132,844.0708
F-stat. *p* value	0.0000	0.0000	0.0000

*Note:* Standard errors in parentheses; averaged data are used; all variables are log-transformed except FDI, population growth and political stability; L.: first lag; H: Hansen test of overidentification; AB: Arrelano–Bond second-order serial correlation test. Zero coefficients and standard errors are due to rounding.

^∗^ *p* < 0.1.

^∗∗^ *p* < 0.05.

^∗∗∗^ *p* < 0.01.

**Table 3:** Long-run two-step system GMM estimates of the effect of breast cancer on LP.

	Model 1	Model 2	Model 3
DALYs	−0.8737^∗∗∗^		
	(0.0911)		

Macroeconomic output	1.0933^∗∗∗^	1.1704^∗∗∗^	1.0732^∗∗∗^
	(0.0704)	(0.1002)	(0.0707)

FDI	−0.0000^∗^	−0.0000^∗∗∗^	−0.0000^∗∗^
	(0.0000)	(0.0000)	(0.0000)

Political stability	0.0120^∗∗∗^	0.0224^∗∗∗^	0.0138^∗∗∗^
	(0.0025)	(0.0026)	(0.0029)

Education	0.7172^∗∗^	0.6317^∗^	0.8078^∗∗^
	(0.2872)	(0.3608)	(0.3146)

Population growth	−0.1545^∗∗^	0.0005	−0.1505^∗^
	(0.0736)	(0.0813)	(0.0826)

YLDs		−0.8547^∗∗∗^	
		(0.1097)	

Deaths			−0.8396^∗∗∗^
			(0.0913)

Constant	−12.0177^∗∗∗^	−17.2827^∗∗∗^	−15.3196^∗∗∗^
	(1.4285)	(2.6049)	(1.7166)

Observations	214	214	214

*Note:* Standard errors in parentheses; averaged data are used; all variables are log-transformed except FDI, population growth and political stability. Zero coefficients and standard errors are due to rounding.

^∗^ *p* < 0.1.

^∗∗^ *p* < 0.05.

^∗∗∗^ *p* < 0.01.

**Table 4:** RE regression estimates of the effect of breast cancer on LP.

	Model 1	Model 2	Model 3
DALYs	−0.3591^∗∗∗^		
	(0.0483)		

Macroeconomic output	0.7940^∗∗∗^	0.8228^∗∗∗^	0.8097^∗∗∗^
	(0.0402)	(0.0409)	(0.0393)

FDI	−0.0000^∗∗∗^	−0.0000^∗∗^	−0.0000^∗∗∗^
	(0.0000)	(0.0000)	(0.0000)

Political stability	0.0003	0.0001	0.0002
	(0.0004)	(0.0005)	(0.0004)

Education	−0.1376^∗∗∗^	−0.1503^∗∗∗^	−0.1415^∗∗∗^
	(0.0515)	(0.0494)	(0.0513)

Population growth	−0.0238^∗∗^	−0.0229^∗∗^	−0.0260^∗∗^
	(0.0120)	(0.0114)	(0.0117)

YLDs		−0.3265^∗∗∗^	
		(0.0437)	

Deaths			−0.3751^∗∗∗^
			(0.0474)

Constant	−6.0590^∗∗∗^	−8.1298^∗∗∗^	−7.5748^∗∗∗^
	(0.5310)	(0.6522)	(0.6142)

Observations	1053	1053	1053
No. of countries	47	47	47
Within *R*^2^	0.8674	0.8697	0.8694
Between *R*^2^	0.3310	0.2430	0.3310
Overall *R*^2^	0.2913	0.2107	0.2900

*Note:* Cluster robust standard errors in parentheses; nonaveraged data are used; all variables are log-transformed except FDI, population growth and political stability. Zero coefficients and standard errors are due to rounding.

^∗^ *p* < 0.1.

^∗∗^ *p* < 0.05.

^∗∗∗^ *p* < 0.01.

**Table 5:** FE regression estimates of the effect of breast cancer on LP.

	Model 1	Model 2	Model 3
DALYs	−0.3373^∗∗∗^		
	(0.0478)		

Macroeconomic output	0.7895^∗∗∗^	0.8279^∗∗∗^	0.8059^∗∗∗^
	(0.0397)	(0.0416)	(0.0398)

FDI	−0.0000^∗∗^	−0.0000^∗∗^	−0.0000^∗∗^
	(0.0000)	(0.0000)	(0.0000)

Political stability	0.0002	0.0000	0.0001
	(0.0004)	(0.0005)	(0.0004)

Education	−0.1559^∗∗∗^	−0.1692^∗∗∗^	−0.1592^∗∗∗^
	(0.0515)	(0.0495)	(0.0512)

Population growth	−0.0189	−0.0185	−0.0212^∗^
	(0.0121)	(0.0114)	(0.0117)

YLDs		−0.3179^∗∗∗^	
		(0.0437)	

Deaths			−0.3547^∗∗∗^
			(0.0478)

Constant	−6.0905^∗∗∗^	−8.2233^∗∗∗^	−7.5373^∗∗∗^
	(0.4962)	(0.6498)	(0.5987)

Observations	1053	1053	1053
No. of countries	47	47	47
Within *R*^2^	0.8682	0.8701	0.8701
Between *R*^2^	0.3073	0.2325	0.3093
Overall *R*^2^	0.2686	0.2009	0.2694

*Note:* Cluster robust standard errors in parentheses; nonaveraged data are used; all variables are log-transformed except FDI, population growth and political stability. Zero coefficients and standard errors are due to rounding.

^∗^ *p* < 0.1.

^∗∗^ *p* < 0.05.

^∗∗∗^ *p* < 0.01.

## Data Availability

The data employed by this study are freely available from the websites of the Global Burden of Diseases Study (https://vizhub.healthdata.org/gbd-results/), the World Bank (https://databank.worldbank.org/reports.aspx?source=World-Development-Indicators#advancedDownloadOptions), and the International Labour Organisation (https://rshiny.ilo.org/dataexplorer12/?lang=en%26id=GDP_205U_NOC_NB_A). Readers should please take into consideration when the authors accessed the datasets as outlined in the reference list [27–29].
